# Novel clinical presentation and *PAX6* mutation in families with congenital aniridia

**DOI:** 10.3389/fmed.2022.1042588

**Published:** 2022-12-13

**Authors:** Ruru Guo, Xiaotian Zhang, Aihua Liu, Jian Ji, Wei Liu

**Affiliations:** ^1^Tianjin Key Laboratory of Retinal Functions and Diseases, Tianjin Branch of National Clinical Research Center for Ocular Disease, Eye Institute and School of Optometry, Tianjin Medical University Eye Hospital, Tianjin, China; ^2^Department of Ophthalmology, Nankai University Eye Hospital, Tianjin, China; ^3^Tianjin Key Laboratory of Ophthalmology and Visual Science, Tianjin Eye Hospital, Clinical College of Ophthalmology, Tianjin Medical University, Tianjin, China

**Keywords:** aniridia, *PAX6*, gene mutation, phenotype, genotype

## Abstract

**Purpose:**

To explore the clinical phenotype and genetic defects of families with congenital aniridia.

**Methods:**

Four Chinese families with aniridia were enrolled in this study. The detailed ocular presentations of the patients were recorded. Whole exome sequencing (BGI MGIEasy V4 chip) was used to detect the gene mutation. Sanger sequencing was performed to validate the potential pathogenic variants, and segregation analysis was performed on all available family members.

**Results:**

By whole exome sequencing and Sanger sequencing, three recurrent mutations (c.112del, p.Arg38Glyfs*16; c.299G > A, p.Trp100* and c.718C > T, p.Arg240*) and one novel mutation (c.278_281del, p.Glu93Alafs*30) of *PAX6* were identified. All the mutations were co-segregated with the phenotype in the families. We also observed spontaneous anterior lens capsule rupture in aniridia for the first time.

**Conclusion:**

We report spontaneous anterior lens capsule rupture as a novel phenotype of aniridia and three recurrent mutations and one novel mutation of *PAX6* in families with aniridia. Our results expanded the phenotype and genotype spectra of aniridia and can help us better understand the disease.

## Introduction

Aniridia is a rare, bilateral, congenital panocular disorder that causes complete or partial absence of the iris and iris hypoplasia. The incidence of aniridia ranges from 1 in 64,000 to 1 in 96,000 population, with no known preference for race or sex (1–3). Aniridia can be accompanied by a range of other ocular anomalies, including corneal abnormalities, cataract, ectopia lentis, glaucoma, strabismus, refractive error, ptosis, microphthalmia, foveal hypoplasia, and optic nerve hypoplasia. Non-ocular sensory and neurological deficits can also be present, including hearing difficulties, reduced olfaction, and Wilms tumor, aniridia, genitourinary anomalies, and intellectual disability (WAGR) syndrome (3). About two-thirds of aniridia cases are familial and inherited as an autosomal dominant trait, with complete penetrance and variable expressivity (4, 5). Most aniridia cases are associated with mutations in the pair box 6 (*PAX6*) gene at chromosome 11p13.

*PAX6* was firstly proposed as the causative gene of congenital aniridia in 1991 by positional cloning (6, 7). Human *PAX6* encodes a 422-amino acid transcriptional regulatory protein, which consists of two DNA-binding domains (a paired domain at the NH_2_ terminal, including 128 amino acids, and a homeodomain, including 61 amino acids) separated by a 79-amino acid linker region, and a transcriptional transactivation domain at the COOH terminus rich in proline, serine, and threonine (6, 7). *PAX6* is expressed in early eye structures, the forebrain, the neural tube, and the pancreas and plays a crucial role in the development of the eye and central nervous system (8, 9). The majority of *PAX6* mutations result in null alleles and consequent *PAX6* haploinsufficiency and lead to aniridia. Other ocular abnormalities have also been associated with *PAX6* changes. Isolated foveal hypoplasia has been described in few families with *PAX6* missense mutations [p.Pro76Arg, p.Arg128Cys, p.Gly72Ser (10–12)] or premature termination codon (PTC) mutations [p.Pro346Aspfs*20, p.Tyr354Cysfs*8 (11)]. Microphthalmia and Peters anomaly are associated with several *PAX6* mutations, most of which are missense, while anophthalmia is associated with homozygous *PAX6* variants (13). With the advances in neuroimaging technologies, both anatomical (changes in the anterior commissure, posterior commissure, pineal gland, corpus callosum, optic chiasm, and olfactory bulb) and functional neuro-abnormalities (deficits in olfactory function, cognitive ability, and auditory interhemispheric transfer) have been described in individuals with aniridia (14), highlighting the crucial role of *PAX6* in development of central nervous system. However, the exact phenotype-genotype correlation is still unclear.

In this study, we enrolled four Chinese families with aniridia to explore the genetic defects of congenital aniridia.

## Materials and methods

The study was approved by the ethics committee of Tianjin Medical University Eye Hospital and was in compliance with the regulations of the Declaration of Helsinki. Informed consent was obtained from the enrolled family members. The method of whole exome sequencing and data analysis is described in detail previously (15–17). Briefly, genomic DNA was extracted from venous blood samples according to the manufacturer’s standard procedure (MagPure Buffy Coat DNA Midi KF Kit, Magen, China) and sequenced on MGISEQ-2000 (PE100) using the BGI MGIEasy V4 chip, which contains exons of all human genes and their adjacent ±20 bp introns. Sanger sequencing was then performed to validate the potential pathogenic variants, and segregation analysis was performed on all available family members. The primers for Sanger sequencing are summarized in Supplementary Table 1. Additionally, the structures of the mutant and homomeric wild-type *PAX6* were modeled by the SWISS-MODEL server^1^ and shown using a PyMOL Molecular Graphic system. The *PAX6* PDB file (AF-P26367-F1-model_v1.pdb) was downloaded from the AlphaFold Protein Structure Database^2^ and was used as a template.

## Results

We enrolled four Chinese families with aniridia in this study.

### Family 1

**FIGURE 1:**
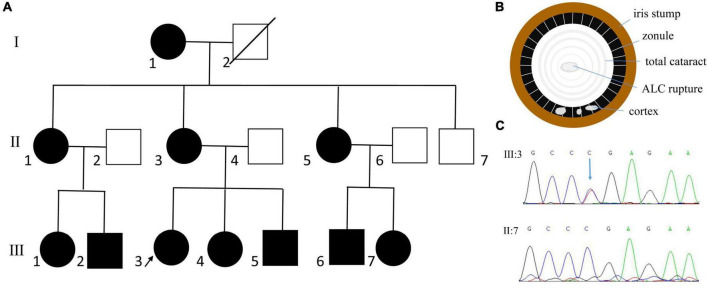
Clinical and genetic evaluation of family 1. **(A)** Pedigree map of family 1. The arrow indicates the proband. The circles and squares represent females and males, respectively. White and black denotes unaffected and affected individuals, respectively. **(B)** Schematic picture showing anterior lens capsule (ALC) rupture in patient with total cataract. **(C)** Sanger sequencing showing a previously reported nonsense mutation of *PAX6* (c.718C > T, p.Arg240*).

The proband of family 1 was a 14-year-old girl. The detailed ocular presentations of the affected members in family 1 are summarized in Table 1. Briefly, 11 individuals in the family were affected and presented with complete aniridia, while four members had nystagmus. All the affected individuals had poor vision (ranging from finger counting to 0.12), and the intraocular pressure (IOP) was high in two patients. Lens abnormalities were detected in eight patients, including cortical cataract, nuclear cataract, total cataract, and lens ectopia. We also observed spontaneous anterior lens capsule (ALC) rupture in two of the three patients with total cataracts, which was never reported before in patients with aniridia. Unfortunately, the ocular anterior segment photographs are not available in this family and a schematic picture was drawn to illustrate the changes (Figure 1).

**TABLE 1:** Ocular manifestations of affected individuals in aniridia family 1.

Individual	Age (year)	Gender	VA (R/L)	IOP (mmHg, R/L)	Aniridia	Nystagmus	Exotropia	Lens
I:1	57	F	0.1/0.1	17.1/19.3	+	+	−	Dense nuclear cataract
II:1	36	F	FC/FC	18.5/14.5	+	−	+	Cortical cataract
II:3	34	F	0.1/0.12	17.8/19.1	+	−	+	pseudophakic
II:5	30	F	0.04/0.02	20.3/20.6	+	−	−	Cortical cataract
III:1	13	F	0.02/0.02	23.7/26.8	+	−	−	Clear
III:2	1.5	M	NA	NA	+	+	−	NA
III:3	14	F	FC/FC	11.8/21.2	+	−	−	Total cataract with spontaneous ALC rupture
III:4	11	F	0.1/0.08	20.7/15.9	+	−	−	Total cataract with spontaneous ALC rupture
III:5	9	M	0.1/0.1	17.3/17.5	+	+	−	Total cataract
III:6	10	M	0.1/0.04	14.1/14.4	+	+	−	Lens ectopia
III:7	3	F	NA	22.4/25.7	+	−	−	Clear

F, female; M, male; NA, not available; FC, finger counting; R, right eye; L, left eye; +, present; −, absent; ALC, anterior lens capsule.

By whole exome sequencing and Sanger sequencing, a previously reported nonsense mutation in *PAX6* (c.718C > T, p.Arg240*) was detected (Figure 1). It was detected in all the affected individuals but not in the unaffected individuals in this family, indicating that the mutation was co-segregated with the phenotype.

### Family 2

**FIGURE 2:**
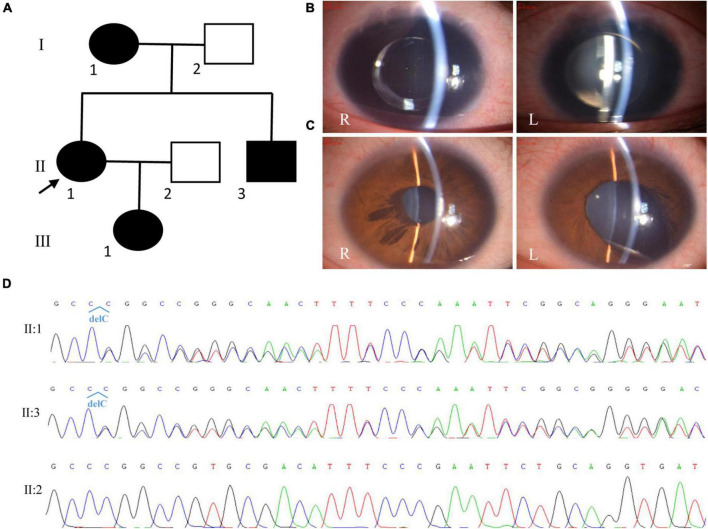
Clinical and genetic evaluations of family 2. **(A)** Pedigree map of family 2. **(B)** Anterior segment photograph showing complete aniridia and the intraocular lens in both eyes of the proband. **(C)** Anterior segment photograph showing ectropion of the iris pigment epithelium in the right eye (R) and iris coloboma in the left eye (L) of the proband’s brother. **(D)** Sanger sequencing showing a previously reported frame-shift mutation in *PAX6* (c.112del, p.Arg38Glyfs*16).

The proband was a 30-year-old woman, and she had complained of blurred vision for more than 20 years. She was diagnosed with aniridia, nystagmus, congenital cataract (posterior polar cataract), and lens ectopia at the age of 15 years. At that time, her visual acuities were 0.12 and 0.1; IOP, 17.8 and 18.1 mmHg; axial lengths, 24.02 and 23.70 mm; central anterior chamber depths, 1.53 and 1.54 mm; flat *K* values, 39.5 and 39.0 D; steep *K* values, 40.5 and 40.0 D; endothelium cell densities, 3677.2/mm^2^ and 3780.2/mm^2^ in the right eye and left eyes, respectively. The patient accepted cataract extraction and intraocular lens implantation in 2007 for the right eye (Morcher GmbH Type 67G, + 22D) and in 2009 for the left eye (Morcher GmbH Type 67G, + 23D). After surgery, her vision improved to 0.2 in both eyes. In 2017, the patient complained of blurred vision again. On presentation, her vision was 0.1 and light perception, and the IOP was 29.1 mmHg in the right eye and 33.2 mmHg in the left eye, respectively. Slit-lamp microscopy (SLM) revealed aniridia and nystagmus, and both eyes were pseudophakic (Figure 2). A fundoscopy examination could not be performed because of severe nystagmus. The patient was given three anti-glaucoma medications to control the IOP. The patient’s mother had similar ocular presentations. The patient’s daughter was 3 months old and had nystagmus and aniridia in both eyes. The patient’s brother was found to have ectropion of the iris pigment epithelium in the right eye and iris coloboma in the left eye (Figure 2). He had normal vision and IOP in both eyes.

By whole exome sequencing and Sanger sequencing, a previously reported frame-shift mutation in *PAX6* (c.112del, p.Arg38Glyfs*16) was detected in the proband, her mother, and her brother (Figure 2). No blood sample was available from her daughter.

### Family 3

**FIGURE 3:**
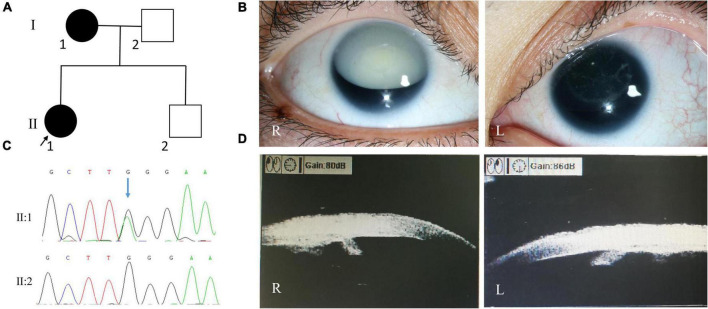
Clinical and genetic evaluations of family 3. **(A)** Pedigree map of family 3. **(B)** Anterior segment photograph showing white cataract of the right eye, cortical cataract of the left eye, and lens ectopia of both eyes in the proband. **(C)** Sanger sequencing showing a recurrent *PAX6* nonsense mutation (c.299G > A, p.Trp100*). **(D)** Ultrasonographic biomicroscopy image showing severe iris hypoplasia in both eyes of the proband.

The proband of family 3 was a 34-year-old woman, and her medical records were reviewed. On presentation, her visual acuity was hand motion and 0.12, and her IOP was 12.7 mmHg in the right eye and 13.5 mmHg in the left eye. SLM revealed nystagmus, aniridia, cataracts (total cataract in the right eye and cortical cataract in the left eye), and lens ectopia in both eyes (Figure 3). In the right and left eyes, the axial lengths were 24.07 and 23.26 mm; central corneal thicknesses, 624 and 636 μm; endothelium cell densities, 3968.8/mm^2^ and 3275.1/mm^2^; flat *K* values, 40.45 and 40.21 D; steep *K* values, 43.12 and 42.15 D; corneal diameters, 11.45 and 11.24 mm, respectively. Ultrasonographic biomicroscopy revealed severe iris hypoplasia in both eyes (Figure 3). The patient accepted sequential cataract extraction of both eyes in 2009, without intraocular lens implantation. After that, the IOPs of both eyes increased (29.5 mmHg in the right eye and 26.6 mmHg in the left eye), and glaucomatous optic neuropathy occurred (cup-to-disc ratio: 0.8). In 2012, the patient accepted Ahmed glaucoma valve implantation for the right eye. One year later, the IOP in both eyes increased again, ranging from 20 to 30 mmHg, under three anti-glaucoma medications. The patient’s mother also had nystagmus, aniridia, cataracts, and glaucoma in both eyes.

Whole exome sequencing revealed a recurrent *PAX6* nonsense mutation (c.299G > A, p.Trp100*; Figure 3). Sanger sequencing revealed that the patient’s mother also carried this mutation, while her father and brother did not.

### Family 4

**FIGURE 4:**
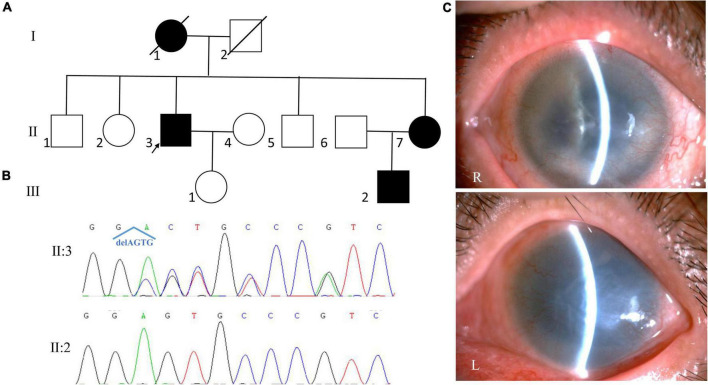
Clinical and genetic evaluations of family 4. **(A)** Pedigree map of family 4. **(B)** Sanger sequencing showing a novel frame-shift mutation of *PAX6* (c.278_281del, p.Glu93Alafs*30). **(C)** Anterior segment photograph of the proband showing corneal neovascularization, aniridia in both eyes, a dense nuclear cataract in the right eye and severe corneal opacity in the left eye.

The proband of family 4 was a 66-year-old man. He complained of bilateral ocular pain for 1 month. He was diagnosed with aniridia when he was 6 years old. On presentation, his visual acuity was hand motion and no light perception, and his IOP was 35.7 mmHg in the right eye and 55.2 mmHg in the left eye. SLM revealed corneal neovascularization, aniridia, and nystagmus in both eyes (Figure 4). A dense nuclear cataract was found in the right eye, and severe corneal opacity was found in the left eye, which made the fundus invisible. Keratoplasty and cataract surgery of the right eye was suggested, but was refused by the patient. The patient was administered anti-glaucoma medications to control the IOP in both eyes and was still under follow-up.

Whole exome sequencing and Sanger sequencing revealed a novel frame-shift mutation in *PAX6* (c.278_281del, p.Glu93Alafs*30; Figure 4). The variant was detected by further Sanger sequencing in all affected patients enrolled in this study (III:2, III:3) but not in the unaffected family members (III:4, III:5, IV:2). The variant was co-segregated with the disease in family members and was not found in NCBI dbSNP, HapMap, 1000 human genome dataset, the database of 100 healthy Chinese adults, and ClinVar, suggesting that the variant may be the pathogenic mutation in this family.

**FIGURE 5:**
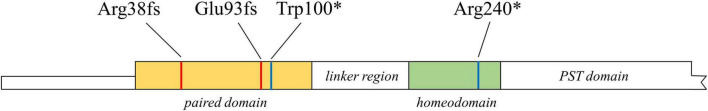
Schematic diagram of the location of the four mutations detected in this study in PAX6 protein. PST, proline, serine, and threonine. *Means a stop codon.

The location of the four mutations detected in this study was highlighted in Figure 5.

### Protein model construction

**FIGURE 6:**
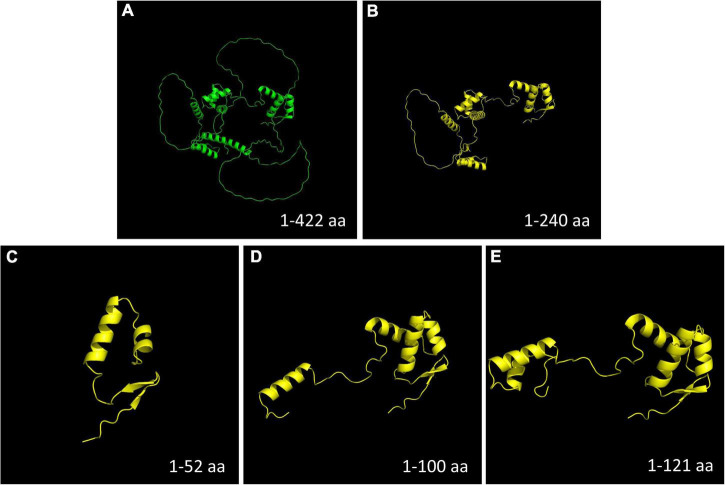
Protein model construction. Compared with the wild-type PAX6 protein, all the four mutations detected in this study were predicted to produce a truncated protein. **(A)** Wild-type PAX6 protein. **(B)** Mutant PAX6 protein of family 1 (c.718C > T, p.Arg240*). **(C)** Mutant PAX6 protein of family 2 (c.112del, p.Arg38Glyfs*16). **(D)** Mutant PAX6 protein of family 3 (c.299G > A, p.Trp100*). **(E)** Mutant PAX6 protein of family 4 (c.278_281del, p.Glu93Alafs*30). Aa, amino acids.

Compared with the wild-type PAX6 protein, all four mutations detected in this study were predicted to produce a truncated protein and interfere with DNA binding (Figure 6).

## Discussion

In this study, we enrolled four families with aniridia and identified three recurrent mutations (c.112del, p.Arg38Glyfs*16; c.299G > A, p.Trp100* and c.718C > T, p.Arg240*) and one novel mutation (c.278_281del, p.Glu93Alafs*30) of *PAX6* by whole exome sequencing. We also found spontaneous ALC rupture in aniridia, which expanded the disease phenotype.

Although aniridia has a spectrum of ocular findings, cataract, glaucoma and keratopathy are main reasons for visual compromise in such patients. Cataracts may develop at any age and are usually progressive. The common morphological types of cataract include posterior polar, posterior subcapsular, and total cataract. Cataract surgery in aniridia is challenging, because of the poor ocular surface, corneal opacification, friable capsule and lens dislocation (18). Iris prosthesis implantation can also be considered in cataract surgery, although several complications may occur during or after the surgery. Glaucoma occurs in 46–70% of cases (19). Several possible mechanisms, including maldevelopment of the anterior chamber angle, absence of Schlemm’s canal, and obstruction of the angle with a shapeless, homogenous avascular tissue, are implicated in the development of glaucoma (18). Although medical therapy remains the first line of treatment, most patients with glaucoma eventually require surgical therapy, such as goniotomy, trabeculotomy, trabeculectomy, glaucoma drainage devices implantation and cyclodestructive procedures (19). However, the management of aniridic glaucoma remains a challenge. At present, there is a lack of prospective and randomized controlled studies on glaucoma treatment in aniridia, and the results from the existing studies are inconsistent. Aniridia-associated keratopathy (AAK), characterized by gradual corneal pannus and opacification, is another common cause of progressive vision loss. AAK is resulted from the breakdown of the limbal stem cell niche, impaired wound healing, and neural deterioration and can occur as early as 2 years of age (18). The management of AAK depends on its severity. In the early stages, supportive treatment includes artificial tear fluid without preservatives and autologous serum are generally recommended. When the keratopathy affects the visual axis, surgery intervention is often needed. Penetrating keratoplasty tends to fail eventually, owing to the underlying limbal stem cell deficiency. Even procedures attempting to restore or improve the limbal stem cell niche, such as amniotic membrane transplantation, limbal or keratolimbal allograft transplantation, cultivated limbal epithelial transplantation, cultivated oral mucosa epithelial transplantation, have a risk of surgery failure (18, 19). Keratoprosthesis is an option in treatment of end-stage AAK. Boston type 1 keratoprosthesis is reported to have good anatomical and functional long-term results in patients with AAK (20). Obviously, an appropriate individualized treatment regimen should be determined for patients with aniridia, in order to achieve the best chance of postoperative success with minimized complications.

According to the Human Gene Mutation Database (HGMD),^3^ over 750 *PAX6* mutations were reported, with predicted premature truncations being the most common *PAX6* mutations. Although so many mutations have been reported, the exact phenotype-genotype correlation of aniridia is not yet clear. However, patients with nonsense or frame-shift mutations that leading to the introduction of a PTC tend to present classical aniridia phenotype (21–23), which is in accordance with our findings. All four mutations detected in this study were truncating mutations, and all four families presented with typical aniridia. It has also been reported that missense mutations are usually associated with milder atypical aniridia phenotypes (21, 22), probably resulting from the prediction that the PAX6 proteins of missense mutations retain some of their functions (24). Nevertheless, a recent statistical analysis of the genotype-phenotype correlations in *PAX6*-associated aniridia revealed that missense mutations were associated with a severe aniridia phenotype similar to the truncating mutations, because some formally missense mutations could disrupt splicing and lead to nonsense-mediated decay (NMD) (25). Obviously, the exact genotype-phenotype correlations remain to be further elucidated.

Phenotypic variability is frequently reported in aniridia, even among members of the same family (13, 22, 26–30). In an analysis of 155 patients with aniridia, complete aniridia was observed in 78% patients, and foveal hypoplasia, cataract, nystagmus, keratopathy, and glaucoma was observed in 85, 80, 78, 58, and 26% patients, respectively (25), which is in accordance with other reports (23, 29). In our study, there were 18 patients with aniridia whose ocular presentations were available. Complete aniridia was the predominant ocular manifestation, which occurred in 17 of the 18 patients (94.4%). Cataract, nystagmus, glaucoma, lens ectopia, strabismus, ALC rupture, keratopathy and iris coloboma occurred in 66.7% (12/18), 55.6% (10/18), 33.3% (6/18), 22.2% (4/18), 11.1% (2/18), 11.1% (2/18), 5.6% (1/18) and 5.6% (1/18) of the patients, respectively. Generally, the incidences of each ocular presentation was comparable to that of other reports (23, 25, 29), except for keratopathy. The percentage of keratopathy was much smaller in our study, this may be resulted from that the enrolled patients were generally younger and aniridia-associated keratopathy is age-dependent (18). Our results also highlight the high phenotypic heterogeneity of aniridia (Table 2). In family 2, the proband presented with complete aniridia, while her brother, who carried the same mutation as the proband, was only found to have ectropion of the iris pigment epithelium in the right eye and iris coloboma in the left eye. In family 1, although all the patients presented with complete aniridia, nystagmus was present in only four members, and the lens abnormalities were variable among the affected individuals. We also observed spontaneous ALC rupture in the patients with aniridia who had total cataracts in family 1, which has not previously been reported. We think that the occurrence of ALC rupture in aniridia is reasonable because it has been discovered that the ALC of patients with aniridia is thinner and more fragile (31–33). Moreover, the intumescence of total cataracts can also facilitate the rupture of the thinner and fragile ALC. This phenotypic variability is not correlated with the location or the nature of the mutation (13). It is still far from clear how *PAX6* mutations translate into variable expressivity among individuals with aniridia from the same or different families (13, 22).

**TABLE 2:** Phenotypes and genotypes of the families with aniridia.

Family	*PAX6* mutation	Nystagmus	Cornea	Lens	Glaucoma	Iris
1	c.718C > T	+	Clear	ALC rupture, cortical cataract, nuclear cataract, total cataract, LE	+	Aniridia
2	c.112del	+	Clear	Posterior polar cataract, LE	+	Aniridia, ectropion of IPE, iris coloboma
3	c.299G > A	+	Clear	Total cataract, cortical cataract, LE	+	Aniridia
4	c.278_281del	+	Corneal neovascularization, corneal opacity	Nuclear cataract	+	Aniridia

LE, lens ectopia; ALC, anterior lens capsule; IPE, iris pigment epithelium; +, positive.

Thus far, most *PAX6* pathogenic mutations are PTC mutations, including nonsense, frame-shift, and splice-site mutations, which are predicted to produce truncated proteins (Figure 6). It is now accepted that mutations leading to PTC located up to 50 bp upstream of the last exon of *PAX6* will give rise to NMD (34). NMD is a process by which abnormal mRNAs resulting from a PTC are degraded before large quantities of truncated proteins are produced (35–37). This is considered a protective mechanism by which abnormal aggregation of truncated protein products in the cell can be prevented. This mechanism illustrates the etiology of aniridia in the families enrolled in this study. All four mutations detected in our study were PTC mutations located before the last 50 bp upstream of the last exon junction and would theoretically activate NMD, which will produce a mutant null allele, resulting in haploinsufficiency (loss of function of one copy). The mRNA transcribed from a single functional allele will lead to a 50% reduction in the PAX6 protein level, which is insufficient to trigger the transcription of its downstream target genes, and consequently, hinder normal eye development and lead to aniridia (38–41).

## Conclusion

For the first time, we report spontaneous ALC rupture in aniridia and detected three recurrent mutations (c.112del, p.Arg38Glyfs*16; c.299G > A, p.Trp100*; and c.718C > T, p.Arg240*) and one novel mutation (c.278_281del, p.Glu93Alafs*30) of *PAX6* in families with aniridia. Our results expanded the phenotype and genotype spectra of aniridia and can help us better understand the disease.

## Data availability statement

The original contributions presented in this study are included in the article/Supplementary material, further inquiries can be directed to the corresponding author.

## Ethics statement

The studies involving human participants were reviewed and approved by the Ethics Committee of Tianjin Medical University Eye Hospital. Written informed consent to participate in this study was provided by the participants or their legal guardian/next of kin. Written informed consent was obtained from the individual(s), and minor(s)’ legal guardian/next of kin, for the publication of any potentially identifiable images or data included in this article.

## Author contributions

WL and JJ designed and supervised the study. RG and XZ collected the data. RG drafted the manuscript. WL, RG, AL, and JJ analyzed the data. All authors read and approved the final manuscript.

## Footnotes

1
https://swissmodel.expasy.org

2
https://alphafold.ebi.ac.uk

3
https://www.hgmd.cf.ac.uk
