# Supplementary material for: Novel clinical presentation and PAX6 mutation in families with congenital aniridia
Source: Front Med (Lausanne). 2022 Dec 13;9:1042588. doi: 10.3389/fmed.2022.1042588 (PMC9792480; doi:10.3389/fmed.2022.1042588)
Supplement: Supplementary file 1 [file Table_1.DOCX]

Table S1 The primers used for Sanger sequencing

| Family | Mutation | Primer (forward) | Primer (reverse) | Fragment length |
| --- | --- | --- | --- | --- |
| 1 | c.760C>T | AGAAAGGTCACTGAATGCCA | GGTGCTGAAACTACTGCTGATA | 593 bp |
| 2 | c.112delC | CTGGTGGTCCTGTTGTCCTTTA | TTACGGTTCATAAACTGTTCCCA | 701 bp |
| 3 | c.299G>A | CAGTAAGTTCTCATACCATTGAAGG | AGAGGACACAGACTAAGAGACAG | 453 bp |
| 4 | c.278_281delAGTG | CAGTAAGTTCTCATACCATTGAAGG | AGAGGACACAGACTAAGAGACAG | 453 bp |
